# Techno-economic assessment of microbial limonene production

**DOI:** 10.1016/j.biortech.2019.122666

**Published:** 2020-03

**Authors:** Chenhao Sun, Constantinos Theodoropoulos, Nigel S. Scrutton

**Affiliations:** a Future Biomanufacturing Research Hub, Manchester Institute of Biotechnology, Department of Chemistry, University of Manchester, Oxford Rd, Manchester M13 9PL, United Kingdom; b Future Biomanufacturing Research Hub, Department of Chemical Engineering and Analytical Science, University of Manchester, Oxford Rd, Manchester M13 9PL, United Kingdom

**Keywords:** Techno-economic analysis, Biorefinery, Terpene, Gas-stripping, Minimum selling price

## Abstract

To satisfy the growing demand for limonene, novel pathways for microbial production of limonene have been sought. A techno-economic analysis is carried out for one such process producing limonene from sugar at an industrial plant scale to assess potential economic viability. A conceptual design of the process is developed, in which a gas stripping-solvent scrubbing method is chosen for recovering limonene from bioreactors based on consideration of payback time and process operability. Minimum limonene selling prices are estimated over a range of fermentation productivity based on the calculation of net present value using discounted cash flow method. Under 45% of the maximum theoretical yield, the selling price reaches $19.9/kg, which could be competitive with established production processes when fermentation productivity is above 0.7 kg/(m^3^·h). Reduction of cost could be realised through improvement of microbial strains, utilisation of cheaper feedstocks, reduction in capital investment and strategic business planning.

## Highlights

• A novel microbial limonene production process at industrial plant scale is designed.
• Limonene is recovered by a new combined gas-stripping/solvent-scrubbing method.
• Multi-parameter techno-economic analysis is conducted to assess economic viability.
• Minimum selling price is $19.9/kg as limonene productivity reaches 0.7 kg/(m^3^·h).
• Uncertainties of parameters used in TEA were addressed by sensitivity analysis.

## 1 Introduction

Limonene is one of the most frequently occurring natural monoterpenes found in the plant kingdom. It is the main component of essential oils extracted from citrus fruit rinds (WHO, 1990). Its applications as a flavour and fragrance additive in food products, cosmetics, household cleaning products and textiles are well known (Ali et al., 2015). Other applications of limonene include use as a solvent, a feedstock for fine chemicals, a precursor for polymeric biomaterials and as an active ingredient in medicine (Ciriminna et al., 2014).

Nowadays, most limonene is produced from citrus rind, a major by-product generated during fruit processing in citrus juice industries (Crawshaw, 2001). Limonene-containing essential oil is first extracted from the flavedo layer of citrus rinds by methods such as cold pressing or hydro-distillation. Limonene with higher purity can then be obtained from the essential oil using vacuum fractional distillation or column chromatography methods (Amanzadeh et al., 2006). As a result of the growing demand for green chemical substitutes, functional derivatives, as well as emerging novel medicinal and dietary applications, global limonene production is increasing at a rapid rate and is predicted to exceed 65 kt by 2023 (Global Market Insights, 2016, John et al., 2017).

Currently, the citrus juice industry ensures a continuous feedstock supply for the production of limonene. However, some of the major citrus fruit producers, especially in Asia, are more interested in whole fruit markets rather than the juice industry. For example, 95% of oranges in China are consumed as whole fruits, in sharp contrast to 44% in Brazil (Axelsson et al., 2016). The difficulty of citrus rind recycling in these regions has constrained the scale of local limonene production, despite the high volume of citrus fruits produced annually. Also, citrus juice industries, especially in developing countries, lack appropriate infrastructure and technology to support on-site recycling of large quantities of citrus waste (Mahato et al., 2018). Consequently, most of the citrus rinds are disposed of in landfills rather than used for limonene extraction. In general, limited capacity and the spatial variety of agriculture-based production poses a major challenge to meeting demands for increased limonene supply.

In order to ensure a stable and sustainable limonene supply, much research effort has been devoted to the development of biotechnological methods of limonene production to complement current production systems. This has been realised by expressing limonene synthases derived from plant species as well as precursor pathways (i.e. MEP and MVA pathways) in model hosts such as *E. coli* (Alonso-Gutierrez et al., 2013, Willrodt et al., 2014, Zebec et al., 2016). Microbial production no longer relies on the citrus industry to provide biomass feedstocks, as microbes are capable of converting a broad range of renewable raw materials to limonene (Jongedijk et al., 2016). Availability of renewable feedstock and advances in genetic engineering make microbial methods of limonene production a potential alternative to established production methods.

Nonetheless, microbial production of limonene is at a relatively low technology readiness level (TRL) and has not gone beyond bench-top scale. Despite numerous research studies on the creation of engineered cells with enhanced performance, the highest fermentation productivity achieved in bench-top bioreactor systems is around 0.02 kg/(m^3^·h), with a glucose-to-limonene yield equivalent to 1% of the theoretical maximum value (Willrodt et al., 2014), to the best of our knowledge. Such productivity and limonene yield apparently lie outside the feasible region for a fermentation process that produces compound with bulk applications, thus the potential future commercial viability of fermentation production of limonene is still uncertain.

The transition of forefront technologies to commercial products is known to be an expensive, time-consuming and risky endeavour. Rational decisions on further investment in process and research development need to be based on techno-economic analysis (TEA). TEA estimates probable production and capital cost of a given process at commercial scale so as to generate a quantitative description of the process’s economic viability (Markham et al., 2016). In biorefineries, TEA based on comprehensive (bio)process- and plant-level models is frequently carried out for process optimisation, scaling-up, combining different technologies into integrated bioprocesses (Binns et al., 2012, D’Angelo et al., 2018, Theodoropoulos et al., 2019, Vlysidis et al., 2011) as well as for optimal scheduling and distribution purposes (Angeles-Martinez et al., 2018). Due to the relative novelty of microbial production of limonene, there have been very few studies to date on a comprehensive TEA analysis of limonene production, although estimates for sugar-based production costs of limonene and cost distribution have been provided in a series of terpene case studies (Wu and Maravelias, 2018). Hence, this work aims to perform TEA to assess the economic potential for the microbial production of limonene using molasses, which is a common byproduct from sugarcane and sugar beet in a raw sugar refinery.

Due to the lack of information on the exact process configurations, parameters and applicable assumptions in the early stage of process development, TEA is limited to providing a ballpark estimate of the investment for a hypothetical limonene manufacturing plant. Nonetheless, the TEA conducted at this level offers a useful comparison between different process alternatives. Moreover, it highlights the gap between early-stage research and commercialisation, so that further process research and development could be directed in the most impactful direction.

## 2 Materials and methods

In this work, the following tasks were performed: conceptual design of 100 m^3^ batch fermentation processes for potential configuration options based on literature survey and bench-scale experiments; construction of corresponding plant-level models using MATLAB and Aspen Plus®; calculation of capital and production costs, followed by preliminary economic evaluation based on payback time method, to enable selection of a feasible process configuration; calculations of discounted cash flow based on the investment estimate of the project and establishment of minimum limonene selling prices (MLSP) for individual scenarios; and sensitivity analysis to address design uncertainties on the economic performance of the process (Tao et al., 2017).

### 2.1 Process technology routes and flow diagrams

The inside battery limit (ISBL) limonene manufacturing plant consists of three core unit operations: fermentation, product recovery and purification. For a large-scale industrial microbial fermentation unit capable of producing around 500–1000 tonnes of limonene per year (i.e. roughly 1–2% of the limonene market potential by 2023), a working volume of 100 m^3^ is assumed (Meyer et al., 2017). Limonene produced during fermentation is constantly removed from culture broth and captured by dodecane (which acts as an absorbent/extractant). Subsequently, limonene can be purified to technical grade (96 wt%) by distillation (Wu and Maravelias, 2018). Note that each batch lasts for 50 h, followed by a 5-hour-long downtime for refilling and discharging the reactor. To eliminate the downtime for continuous operation of downstream processes, the fermentation unit is designed with 11 parallel batch stirred tank bioreactors (STBRs), each with 10 m^3^ working volume. A new batch is commenced every five hours, so that 10 reactors are in operation at any given time to provide a constant output to downstream units. By employing this setup, we can assume the limonene productivity of the whole fermentation unit to be constant, despite the productivity in individual reactors which will vary as a result of changing cell growth phase, nutrient availability, pH and other factors. From a biorefinery complexity point of view, this one-platform (terpene synthesis from C6 sugar) biorefinery involves one feedstock (sugar crops), one product (limonene) and three processes (aerobic fermentation, generic separation and distillation). Their feature complexity indices are ‘7′ (platform), ‘1′ (feedstock), ‘2′ (product) and ‘1/2/1′ (processes), leading to an overall complexity index of 14 (Jungmeier, 2014).

Next, we need a clear definition of the product recovery unit operation in order to identify all the major instruments and process streams to enable cost estimation for the proposed process. Practical *in situ* recovery techniques have already been developed for recovering other volatile organic compounds (VOCs), such as acetone, butanol and ethanol (Outram et al., 2016). Liquid-liquid extraction (LLE) is a widely used technique, where the fermentation broth is mixed with an immiscible organic extractant (e.g. dodecane, diisononyl phthalate). This method exploits the differences in solubilities of the VOC in the aqueous and organic phase (Roffer et al., 1987). The product released into the extracellular environment would preferably partition into the organic phase, from which the product can be further purified. The other method is gas-stripping (GS), which exploits the volatility of the product (Ezeji et al., 2004). This process involves the removal of the product by gas passing through the fermentation broth (this is typically aerating gas for aerobic fermentation). The eluted product can then be recovered from the gas stream by solvent scrubbing.

**Fig. 1:**
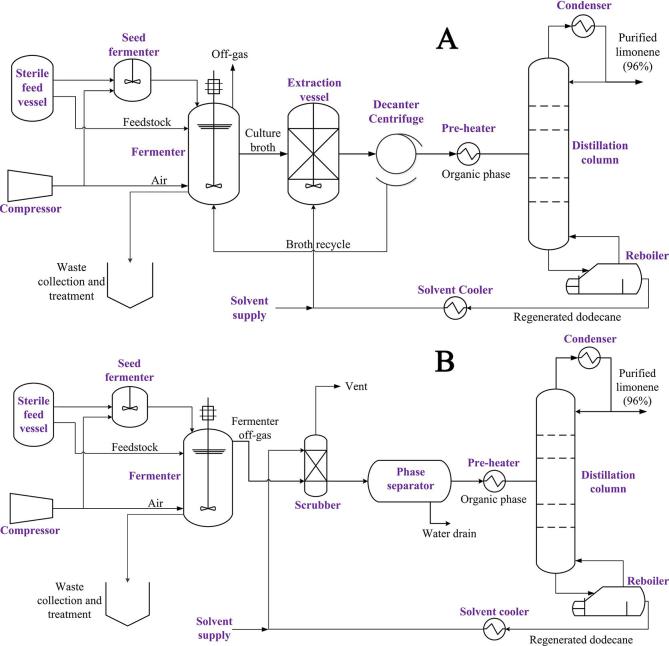
Process flow diagrams for the production process with LLE recovery method (A) and GS recovery method (B).

Given the volatile nature of limonene (vapour pressure ~260 Pa @25 °C) as well as its insolubility in water (<10 mg/L water), both recovery techniques seem compatible with the fermentation process. Hence, two process configurations based on the different recovery methods are proposed accordingly. Both process flow diagrams are illustrated in Fig. 1 (for simplicity, pumps, holding tanks and parallel STBRs are not shown

In the first configuration (Fig. 1, A), a portion of culture broth is continuously discharged into an extraction vessel where it is mixed with dodecane to allow partitioning of limonene into the organic phase. Meanwhile, aerating gas is competing with the liquid discharge stream for limonene removal, resulting in reduction in overall recovery. The organic phase is subsequently separated from culture broth by a centrifugal decanter and sent to the distillation column for limonene purification, while the broth is circulated back to the fermenter. The second configuration (Fig. 1, B) is different from the first one in that limonene is removed from the culture broth entirely by an aerating gas stream. The fermenter off-gas and dodecane is then passed through a scrubber in counter-current flow to remove limonene from the gas. Here we have considered that the organic mixture contains only limonene and dodecane. As a first step, base case scenarios were formulated based on both proposed process concepts so a preliminary comparison of their economic performance could be established.

### 2.2 Design basis and assumptions

The base case assumptions for the proposed process are listed in Table 1. The STBRs were designed to operate in fed-batch mode with an initial glucose concentration of 50 kg m^−3^ and a subsequent feed flow rate which is dependent on the limonene production rate. Given that fed-batch fermentation is a dynamic process with time-dependent outputs (e.g. working volume, product concentration and rate of recovery), simulation of limonene production was performed in MATLAB 2019a. The fermenter model was simulated under the assumption that four simultaneous biochemical reactions take place in the STBRs: biomass synthesis (Eq. (1)), limonene synthesis (Eq. (2)), respiration (Eq. (3)) and acetic acid synthesis (Eq. (4)) (Wu and Maravelias, 2018):

(1) $$C_{6}H_{12}O_{6}\to 6CH_{1.77}O_{0.49}+0.69H_{2}O+{1.185O}_{2}$$

(2) $$C_{6}H_{12}O_{6}\to 0.4286C_{10}H_{16}+1.714CO_{2}+{2.517H}_{2}O$$

(3) $$C_{6}H_{12}O_{6}+6O_{2}\to 6CO_{2}+{6H}_{2}O$$

(4) $$C_{6}H_{12}O_{6}+O_{2}\to 2C_{2}H_{4}O_{2}+2CO_{2}+{2H}_{2}O$$

**Table 1:** Base case process assumption for limonene biomanufacturing process.

Parameters	Value	Source
Limonene productivity (kg/(m^3^·h))	0.1–1	assumed
Limonene recovery (fermenter) (% w/w)	optimisable	–
Limonene recovery (scrubber) (% w/w)	optimisable	–
Operating Mode	Fed-batch	assumed
Target limonene purity (wt %)	96	assumed
Process energy utilisation efficiency (%)	60	assumed
Initial reactor working volume (m^3^)	100	assumed
Batch time (h)	50	assumed
Down time (h)	5	assumed
Initial glucose concentration (kg/m^3^)	100	assumed
Glucose concentration in the feed (kg/m^3^)	800	assumed
Glucose distribution to limonene (w/w)	0.45	assumed
Glucose distribution to acetic acid (w/w)	0.05	assumed
Biomass synthesis/respiration carbon split ratio	0.47	(Wu and Maravelias, 2018)
Specific power input (i.e. P/V ratio) (W/L)	1.5	(Benz, 2008)
Limonene recovery (distillation)	99	assumed
Operating temperature and pressure in the fermentation and product recovery unit operation	30 °C, 1 atm	assumed

Vapour-liquid equilibrium (VLE) of limonene in culture broth was determined at atmospheric pressure based on our bench-top test results. Aeration rates were set to satisfy the minimum oxygen consumption rate required to sustain metabolism, meanwhile ensuring that target limonene recovery can be achieved (Garcia-Ochoa and Gomez, 2009). The model generated time-profiles for process variables, including the rate of limonene removal and limonene mass fractions in both aqueous and gas phase throughout the course of batch fermentation. Averaged stream outputs were subsequently calculated for use by the downstream continuous units.

Downstream separation of limonene from dodecane was simulated in Aspen Plus under the license of Aspentech. The UNIFAC group contribution method was chosen as the thermodynamic package as its simulation resulting in the binary interaction of limonene and dodecane (which appears to form an ideal mixture) matches well with our experimental data. Some heuristics were used in setting the operating parameters to avoid unrealistic design and simulation faults (e.g. the operating pressure of reboiler was chosen to ensure that the boiling point of the bottom product is below that which can be easily obtained with MP plant steam). The combined model produced material and energy balance information for both processes, which was subsequently used for estimating their respective utility costs and sizing the vessels and equipment.

Low productivity is one of the major hurdles which prevent a fermentation process from achieving commercial success. It is crucial to understand the influence of productivity on the process economy, so that target productivity levels could be set for future research development. In addition, both technology routes have a trade-off between the recovery of limonene and economic performance. For instance, raising the recovery rate of limonene in fermenter/absorber could lead to higher limonene production, subsequently higher revenue, but would require a more costly downstream process and higher operating expenses (and vice versa). Thus, the simulation was performed over a range of limonene productivity for both process configurations. And for each productivity scenario studied in economic analysis, the model would ensure that an optimal product recovery goal is identified, which can yields the lowest possible payback time or minimum selling price (terms are explained in Section 2.4).

### 2.3 Cost estimation

The fixed capital investment (FC) is estimated using the factorial method, where the total capital cost was related to inside battery limit (ISBL) cost by factors that account for indirect costs (such as offsite investment, engineering and design costs, and contingency charges). ISBL cost was further related to the purchased equipment by factors accounting for other field costs (e.g. piping and instruments, installation, plant erection, civil work and building construction). The equipment purchase costs were in turn correlated to equipment sizes. Correlations used for estimating the purchase costs of most equipment as well as total capital costs were adapted from a chemical engineering handbook (Sinnott and Towler, 2009) and published design report (KLM Technology Group, 2014). Equipment costs calculated based on historical correlations were adjusted to 2019 USD using Chemical Engineering Plant Cost Index (CEPCI) (Eq. (5)):

(5) $${\mathrm{Ce}}_{2018}={\mathrm{Ce}}_{A}\times {\mathrm{CEPCI}}_{2018}/{\mathrm{CEPCI}}_{A}$$

where *Ce* standards for the equipment purchase cost, and the subscript stands for the specific year in which the value is estimated. *CEPCI2018* equals 603.1. The method, although originally derived from chemical process industries, is assumed to be applicable to biochemical plants as they often resemble conventional processing units. In cases where cost correlations are not available, cost data were obtained from vendors.

The variable costs of production (VCOP) sum up the spending on raw materials, consumables, utilities and other costs that are proportionate with the plant outputs. Costs of glucose, dodecane, ammonium sulphate and phosphate salts (as used in M9 medium) were set according to the average wholesale prices from a range of vendors. Meanwhile, NaOH (1 M solution) was employed as a pH regulating agent to neutralise any acetic acid generated by *E. coli* during fermentation. Utility costs were estimated according to the unit prices that correspond to UK conditions. Key unit prices used in VCOP estimation are summarised in Table 2. Fixed costs of productions (FCOP), including rent, insurance charge and maintenance, were estimated from capital investment, in addition to labour cost (with £25,000 per shift per year) and related overhead charges. Working capital (WC) covering the plant start-up cost, inventory and accounts receivable/payable were estimated from VCOP and FC.

**Table 2:** Major prices used for the base cases in the analysis.

Parameters	Value	Sources
Glucose ($/kg)	0.506 (range: 0.40–0.61)	Vendor quotations
(NH_4_)_2_SO_4_ ($/kg)	1.5	Vendor quotations
Mineral medium (excluding (NH_4_)_2_SO_4_) ($/m^3^)	4.8	Vendor quotations
NaOH ($/kg)	0.396	Vendor quotations
Process cooling water (20 °C inlet, 50 °C outlet) ($/tonne)	0.44	Internal cost data
Steam (15 bar, 198 °C) ($/tonne)	15.84	Internal cost data
Electricity ($/kW-h)	0.17	Department for Business, Energy and Industrial Strategy of the UK
Dodecane ($/kg)	2.17 (range: 1.73–2.60)	Vendor quotations

### 2.4 Economic analysis methods

The base case analysis was estimated for a plant designed for a 15-year project, which is depreciated using the straight-line method over a 7-year period (i.e. half of the project life). We first used the payback time method to evaluate the relative viability of the two proposed process configurations (Eq. (6)).

(6) Payback time=(FC+WC)/(Revenue-FCOP-VCOP)

This method assumes all investment is made in year zero and revenues begin immediately. Revenue was calculated by taking the limonene unit price at $14.04/kg, which is the average price quotes obtained from suppliers for 96% pure limonene.

Based on the estimated payback times and process considerations, the more viable process was chosen and set as the base case for further economy analysis using discounted cash flow method (DCF). DCF analysis finds the minimum limonene selling prices (MLSP) at which limonene must be sold in order to generate a net present value (NPV) of zero for a specified hurdle rate by the end of the project (Eq. (7)).

(7) $$\text{NPV}=\sum_{N=1}^{N=t}\left[{\mathrm{CF}}_{N}/{\left(1+i\right)}^{N}\right]$$

where *i* is the hurdle rate and *N* is the project life in years. *CF_N_* is the cash flow in the *N*th year.

Major financial assumptions used in TEA are summarised in Table 3. The analysis is focused on finding the fermentation productivity at which MLSP is within a reasonable range of limonene selling prices in the current market. Sensitivity cases are developed from the base case for the impact of economic assumptions on project financials.

**Table 3:** Financial assumptions for TEA.

Item	Value
Project start	2019
Project length (year)	15
Plant depreciation schedule	Proportional
Depreciation period (year)	Half of project length
Debt ratio	0.4
Equity ratio	0.6
Cost of debt	0.08
Cost of equity	0.15
Overall hurdle rate	12.2%
Inflation	2%
Construction time (year)	2
% of FC spent in year 1	30
% of FC spent in year 2	70
Startup time (year)	1
% of FCOP during startup	100
% of VCOP during startup	50
% of VCOP during startup	50
Plant salvage value	0
Corporate tax	19%

## 3 Results and discussion

### 3.1 Selecting a suitable process configuration

**Fig. 2:**
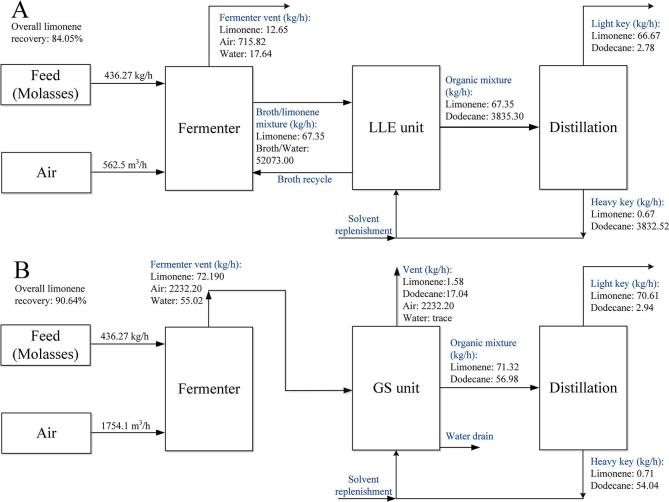
Example of process balances for the production process with LLE recovery method (A) and GS recovery method (B), calculated for limonene productivity of 0.8 kg/(m^3^·h)).

As previously discussed, TEA was first conducted using the payback time method to compare the profitability of the limonene processes using GS and LLE for limonene recovery, respectively. Cost estimation was performed over a range of limonene productivities (i.e. 0.1 to 1 kg/(m^3^·h)). Results are only shown for simulation with productivity of 0.6 kg/(m^3^·h) or higher, as with lower productivity both processes would operate with very small or even negative annual cash flow, resulting in unrealistic payback times (Table 4). The breakdown of the ISBL cost and production cost is presented in Table 5. Examples of the material balance constructed for the main process streams of the base cases are shown in Fig. 2.

**Table 4:** Cost summary for both GS and LLE based limonene production process (M$ = 1 million US dollars).

GS	Payback time	Overall limonene recovery	Productivity (kg/(m^3^·h))	FC (*M*$)	WC (*M*$)	FCOP (*M*$)	VCOP (*M*$)	Revenue (*M*$)
	13.31	0.902	0.6	8.32	0.98	1.78	2.55	5.06
	8.48	0.899	0.7	8.40	1.07	1.79	2.85	5.89
	6.30	0.906	0.8	8.58	1.16	1.80	3.21	6.78
	5.05	0.905	0.9	8.73	1.25	1.81	3.51	7.62
	4.25	0.903	1	8.84	1.33	1.82	3.82	8.45
LLE	Payback time	Overall limonene recovery	Productivity (kg/(m^3^·h))	FC (*M*$)	WC (*M*$)	FCOP (*M*$)	VCOP (*M*$)	Revenue (*M*$)
	21.91	87.02	0.6	8.59	1.00	1.80	2.65	4.88
	14.04	85.09	0.7	8.91	1.09	1.82	3.01	5.57
	11.16	84.05	0.8	9.37	1.20	1.85	3.42	6.29
	9.62	82.13	0.9	9.69	1.30	1.87	3.79	6.91
	8.71	81.11	1	10.19	1.40	1.90	4.21	7.59

**Table 5:** ISBL cost and VCOP breakdown. A: Fermentation; B: Recovery; C: Distillation; D: Raw materials and consumables; E: Sterilisation and waste disposal; F: Heating/Cooling; G: Mixing, pumping and compression.

GS	Productivity (kg/(m^3^·h))	A (*M*$)	B (*M*$)	C (*M*$)	D ($/batch)	E ($/batch)	F ($/batch)	G ($/batch)
	0.6	2.78	0.73	0.36	14,586	868	50	2,608
	0.7	2.84	0.70	0.36	16,887	887	54	2,641
	0.8	2.91	0.71	0.36	19,246	909	63	2,884
	0.9	2.98	0.72	0.36	21,548	928	67	2,917
	1	3.04	0.71	0.36	23,850	947	71	2,950
LLE	Productivity (kg/(m^3^·h))	A (*M*$)	B (*M*$)	C (*M*$)	D ($/batch)	E ($/batch)	F ($/batch)	G ($/batch)
	0.6	2.74	0.81	0.44	14,020	851	1,441	2,036
	0.7	2.81	0.87	0.45	16,281	872	1,607	2,220
	0.8	2.88	1.00	0.47	18,541	892	1,890	2,485
	0.9	2.94	1.08	0.48	20,802	912	2,041	2,693
	1	3.01	1.23	0.50	23,062	933	2,322	3,000

**Fig. 3:**
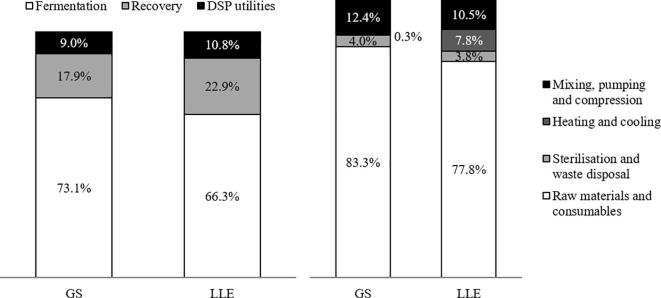
Distribution of ISBL cost (left) and VCOP (right). The percentage is calculated based on the average costs from different productivity cases (productivity range: 0.6 to 1 kg/(m^3^·h)).

The fermentation unit accounts for the largest portion of ISBL plant cost (in proportion to FC) (Fig. 3, left), but has little impact on the relative economic feasibility as its cost stays almost identical for both processes at between $2.7 – 3.0 M (Table 5). In fact, the GS-based process provides notable fixed cost advantage over the LLE-based process owing to its cheaper downstream process. Since the LLE unit deals with transporting, mixing, and centrifugation of liquid streams of significantly greater flow rates than in the GS case, larger vessel sizes and more powerful pumps are required. As a result, the average ISBL cost (for productivity in the 0.6–1 kg/(m3·h) range) of the LLE unit is 40% higher than that of GS recovery unit. Moreover, the GS recovery unit is capable of concentrating limonene in the organic mixture (dodecane) to over 50 wt%, which is far greater than the 2 wt% achieved in LLE unit. This directly results in reduced burden for downstream separation and subsequently a 30% capital cost reduction on the distillation unit.

For VCOP, fermentation-related costs, including the cost of carbon source, sterilisation and waste disposal still dominate the cost structure with approximately 80% of contribution (Fig. 3, right). Electricity usage by mechanical devices is the second largest contributor of production cost at 10.5–12.4%. The GS-based process is relatively more electricity-intensive due to its greater demand for compressed air. On the other hand, with the gas-stripping recovery method providing a much more concentrated limonene distillation feed, the energy expenditure (i.e. steam/cooling water) for the thermal separation of limonene and dodecane is drastically reduced (Table 5). Overall, the GS-based process offers up to 5% VCOP saving, which is favoured by higher limonene productivity.

In addition to ISBL cost and VCOP, the limonene recovery rate is also a critical factor, as it determines the amount of finished product harvested and influences the revenue linearly. In this regard, gas-stripping is also superior to LLE. With the GS method, the optimal limonene recovery is maintained at 90% over the range of productivity investigated; meanwhile, for the LLE method, limonene recovery drops from 88% down to 81% as productivity increases from 0.6 to 1 kg/(m^3^·h). Given the advantages in FC, VCOP and product recovery rate, the gas-stripping method appears to be more economically-attractive than LLE for recovering limonene, as confirmed by its apparently lower payback times (Table 4).

Given the margin of error existing in the conceptual design at the current stage, it may be premature to draw a definite conclusion on the most suitable recovery methods yet. Additional consideration regarding the technical aspect of the process was therefore included. Maintaining axenic conditions of fed-batch fermentation over a long period of time is an important aspect in bioprocesses. The risk of contamination is particularly pronounced in the LLE process which involves the recycling of fermentation broth containing cells and nutrients back to the fermenter. As long as axenic cultivation is a prerequisite for this fermentation system, the recycling loop would require additional re-sterilisation by either steam autoclaving or filtration. However, the flowrate of the recycling stream is in the order of tens of m^3^/h. Sterilisation of a stream with such flowrate can be prohibitively costly for either means. Moreover, it is impractical to continuously replace the active cells lost during sterilisation with new ones. In contrast, the GS-based process that does not require a broth recycling stream offers an obvious advantage in operability and cost-saving. Given both economic and technical considerations, GS is clearly favoured, and is thus chosen as the model method for limonene recovery.

### 3.2 Minimum limonene selling price

**Fig. 4:**
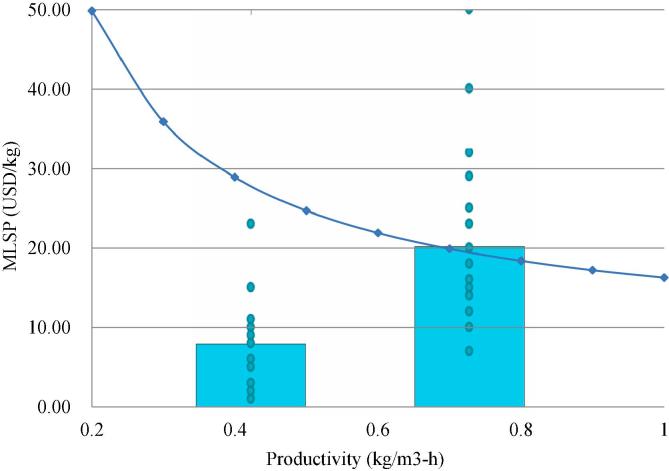
Variation of MLSP over a range of limonene fermentation productivity (connected scatter plot). Averaged upper and lower bound prices for limonene (96% wt) are indicated with bars, with the distribution of prices shown by scatter plots.

Using the calculated capital costs, production costs and financial assumptions stated earlier, MLSP was calculated for the GS case over a range of limonene productivity. Fig. 4 shows the variation of MLSP over a range of productivity. As the productivity increases, the minimum limonene price for an economically feasible scenario declines, eventually reaching $16.29/kg at the productivity of 1 kg/(m^3^·h). However, it should be noted that the market price of limonene likely lies between $7.92/kg and $20.15/kg, meaning that limonene productivity at least needs to reach 0.7 kg/(m^3^·h) (at which the MLSP is $19.92) for the process to be economically viable. Given the current state of technology, a 20-fold improvement in productivity would be necessary. Furthermore, if the aim is to gain a competitive advantage over conventional limonene production, then higher productivity needs to be pursued. This TEA framework for the early stage design and screening of conceptual process alternatives can also be extended to similar bio-based production systems to generate insights into research priorities and help establish the criteria for successful technology implementation.

### 3.3 Sensitivity analysis

To better understand the uncertainties of the base case economics, one-at-a-time sensitivity analysis was conducted for the design variables shown in Table 6. By varying one design parameter between reasonable boundaries at a time, while keeping the other parameters constant, the analysis could quantify the impact of individual variables on MLSP. Variable bounds were chosen based on engineering considerations, as shown in Table 6. During the sensitivity analysis, the plant productivity is maintained at 0.8 kg/(m^3^·h), where the base case MLSP is $18.42/kg.

**Table 6:** List of parameters and their respective bounds for sensitivity analysis.

	Base value	Lower bound	Upper bound	% Variable change
Limonene yield from substrate (kg per kg carbon source)	0.45	0.3	0.5	−33 to 11
Substrate cost ($/kg)	0.506	0.4	0.61	−21 to 21
Plant energy efficiency (%)	60	40	70	−33 to 17
Project length (year)	15	10	20	−33 to 33
Debt ratio (%)	40	30	80	−25 to 100
Cost of equity (rate of return)	0.15	0.1	0.25	−33 to 67
Interest rate	0.08	0.05	0.12	−38 to 50
Total Capital investment (%)	100	80	120	−20 to 20
Fixed cost of production	100	80	120	−20 to 20

**Fig. 5:**
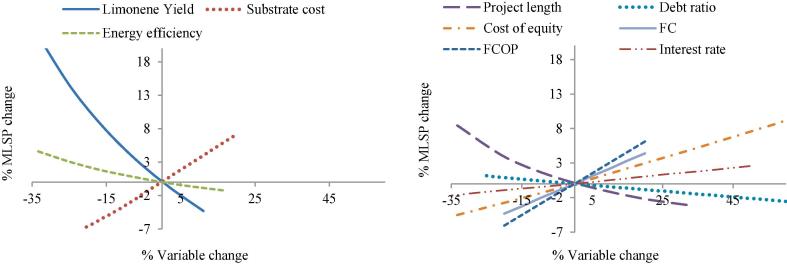
Spider chart for sensitivity analysis for process variables (left) and financial variables (right). The x-axis represents the percentage change in the sensitivity parameters; the y-axis represents the percentage change in MLSP.

**Fig. 6:**
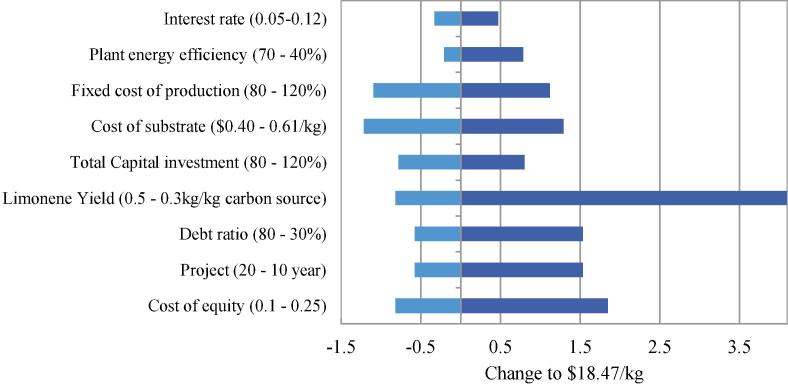
Impact of variable uncertainties on MLSP (base case price: $18.47/kg limonene).

Fig. 5 compares the sensitivity of the various factors on MLSP, where a steeper line indicates a stronger impact. The variation of MLPS due to variable uncertainties is shown in Fig. 6. The variables can fall into two categories: process variables and financial variables. Process variables, including limonene yield, substrate price and energy efficiency, directly govern the production costs. These variables are positively correlated to MLSP apart from substrate price. Limonene yield represents the weight-to-weight conversion of substrate into product, so further increase in yield is equivalent to reduction in substrate consumption under fixed productivity. In the light of the predominance of substrate cost in limonene production, any change in substrate price and substrate yield makes an immediate impact on MLSP. For instance, switching the carbon substrate from molasses to crude glycerol (80% pure, $0.115/kg) would lead to a reduction in MLSP of almost 30% (from $18.42/kg to $13.3/kg). Improvement of energy efficiency is of less significance because utility cost occupies only a small proportion of the direct production cost. Effective cost reduction can be obtained by development of bacterial strains with enhanced genetic characteristics to boost limonene yield and allow the utilisation of cheaper substrates. Process economics can also benefit from a more energy efficient process design of downstream recovery and purification process.

Financial variables are directly related to net cash flow, hurdle rate and other financial indicators that reflect the quality of business operation. Most of the variables have noticeable impact on MLSP, except for debt ratio and interest rate. Amongst the cost drivers, FCOP, FC and cost equity are positively correlated to MLSP, while the project length is negatively correlated to it. The financial variables can be improved to bring further economic advantage by means such as reduction of FCOP by cost-saving methods (e.g. reduction in labour cost due to automation) or extension of useful plant life. Such strategies, however, would not simply be within the scope of research and engineering, but rather a matter of planning and decision-making in business operation.

## 4 Conclusions

TEA was performed for an early-stage biotechnological pathway to produce limonene from sugar via aerobic fed-batch fermentation. Gas-stripping was chosen over liquid–liquid extraction for recovering limonene from fermentation broth. By improving the productivity from 0.2 to 1 kg/(m^3^·h), the MLSP for the selected technology route is projected to fall by 70% to $16.29/kg. Moreover, sensitivity analysis identifies main cost drivers for the process, including limonene yield, substrate price, capital investment, project length and hurdle rates. These factors should be improved in a synergistic manner for a more economically viable process.

## CRediT authorship contribution statement

**Chenhao Sun:** Conceptualization, Investigation, Software, Writing - original draft. **Constantinos Theodoropoulos:** Supervision, Writing - review & editing. **Nigel S. Scrutton:** Supervision, Writing - review & editing, Funding acquisition.

## Declaration of Competing Interest

The authors declare that they have no known competing financial interests or personal relationships that could have appeared to influence the work reported in this paper.
